# Arbitrage-free smile construction on FX option markets using Garman-Kohlhagen deltas and implied volatilities

**DOI:** 10.1007/s11147-022-09189-9

**Published:** 2022-09-18

**Authors:** Matthias Muck

**Affiliations:** Chair of Banking and Financial Control, University of Bamberg, Kärntenstr. 7, 96045 Bamberg, Germany

**Keywords:** FX options, Smile construction, Delta, No-arbitrage, Derivatives, G11, G13

## Abstract

This paper addresses arbitrage-free FX smile construction from near-term implied volatility dynamics proposed by Carr (J Financ Econ, 120(1), 1–20, 2016). The approach is directly applicable to FX option market conventions. Prices of market benchmark contracts (risk reversals and butterflies) are identified as the roots of a cubic polynomial and ATM-volatility can be matched by construction. Implied volatilities are computed with respect to (non-premium adjusted) option deltas. The approach is compared to the Vanna Volga Approach, which does not guarantee arbitrage-free prices. An empirical application to a normal and a stress scenario demonstrates that arbitrage-free implied volatilities coincide with those from the Vanna Volga Approach when prices are interpolated between the $\Delta$25-call and $\Delta$25-put options. Differences are observed when implied volatilities are extrapolated to the wings. Empirically, these differences are particularly relevant in a stress scenario during the Coronavirus crises (2020).

## Introduction

The pricing of currency options is of major concern to academics and practitioners as they convey the market view on the cost of hedging foreign exchange (FX) exposures with structured products. As discussed by Bakshi et al. (2008) and Branger et al. (2021), their prices include information on international stochastic discount factors and, thus, international risk premiums. Moments of the risk neutral probability distribution can also be estimated by setting up buy-and-hold option portfolios assuming that options with a continuum of strike prices are available^1^. Building on this observation, Gao et al. (2018) and Gao et al. (2019) construct indices from option prices measuring tail risk in the risk neutral distribution of the underlying asset.

This paper addresses arbitrage-free smile construction on FX option markets, which are subject to a number of distinctive market conventions. First, currency option prices are quoted in terms of implied volatilities, which are calculated from the Garman and Kohlhagen (1983) model, that is, a variant of the Black and Scholes (1973) model. Second, implied volatilities are given with respect to option deltas (and not strikes). Third, information on implied volatilities is provided for a few benchmark contracts per maturity including at-the-money (ATM) volatilities, risk reversals, and butterflies. Implied volatilities for other contracts have to be extra- or interpolated. To construct arbitrage-free FX smiles the approach of Carr and Wu (2011, 2016) is considered. This approach develops a general framework and different process specifications for the implied volatility as special cases. The focus of Carr and Wu (2011, 2016) is on the dynamics of the entire volatility surface, which is identified by a few model parameters. In contrast, this paper looks at the smile construction for single maturities and smile inter- and extrapolation. For a special case similar to the “Proportional Volatility Dynamics” in Carr and Wu (2016), arbitrage-free FX smiles are determined with respect to directly observable put option deltas. The approach is compared to the related Vanna Volga Approach considered by Lipton and McGhee (2002). The Vanna Volga Approach is applicable when only a few benchmark prices exist but it does not guarantee arbitrage-free smiles. As it is also discussed in detail in Castagna (2010), it assumes a constant volatility for all strikes and adds a premium for hedging vega, vanna, and volga model risk. Therefore, comparing both approaches also indicates to which extent the Vanna Volga Approach yields arbitrage-free FX option prices.

The paper makes the following contributions to the literature. First, no-arbitrage conditions for currency option implied volatilities are determined within the framework of Carr and Wu (2016) with respect to (non-premium adjusted) deltas instead of strikes. For the “Proportional Volatility Dynamics”, arbitrage-free implied FX volatilities are given by the roots of a cubic polynomial. Notably, this cubic polynomial has a symmetry property. By virtue of this property prices of the benchmark contracts risk reversal and butterfly are identified from its smallest positive and its largest negative root. Moreover, ATM-volatilities can be matched by constraining the parametrization of the model appropriately.

Second, the Arbitrage-Free Approach is applied to market data. A normal and a stress scenario are considered for implied volatilities of options on the EUR exchange rate. The stress scenario is a trading day during the height of the Coronavirus crisis in April 2020 while the normal scenario is a trading day during October 2019. Results are compared to those of the Vanna Volga Approach. Interpolation between $\Delta$25-call and $\Delta$25-put implied volatilities yield similar results in both approaches. Differences are observed when volatility smiles are extrapolated to the wings. These differences are more substantial for the stress scenario than for the normal one.

Third, comparative statics suggest that moments of the near-term dynamics of implied volatilities are linked to the shape of the volatility smile. The near-term volatility of implied volatility is positively related to the steepness of the smile. Therefore, to ensure that arbitrage-free implied volatilities in the wings exist, it must not be too large. The near-term correlation between implied volatility and the exchange rate drives the smile skewness. Similar to the near-term volatility, it has little impact on the level of the smile. The level of the smile is mainly related to the expected return of implied volatility and the variance of the forward exchange rate. This observation also motivates the identification of the near-term expected return from the other parameters. Empirically, volatilities of implied volatilities are particularly high for short-term options and the exponential rate of decay is not constant with respect to expiry as specified in Carr and Wu (2016). Furthermore, the near-term correlation also depends on the expiry considered.

The paper is related to two main strands in the literature including the literature on smile/surface construction and on currency option pricing. The literature on smile/surface construction includes the papers by Carr and Wu (2011, 2016) and the contributions on the Vanna Volga Approach discussed above. Moreover, Malz (1997) proposes a parabolic approach to estimate volatility smiles. Wystup (2017) suggests a slice kernel approach, which estimates implied volatilities with respect to deltas directly as well. Other practically relevant procedures for smile construction in the FX market are the stochastic volatility inspired (SVI) approach (Gatheral, 2004) and the application of the SABR model (Hagan et al., 2002). Furthermore, Dumas et al. (1998) suggest implied volatility functions that describe the volatility surface. The literature on pricing models for currency options includes the Black/Scholes approach by Garman and Kohlhagen (1983), the stochastic volatility model by Heston (1993), and the approach by Bates (1996), which addresses jump risk. Branger and Muck (2012) consider the pricing of FX options in the presence of stochastic correlation. Further FX stochastic volatility models are developed, for example, by De Col et al. (2013) and Gnoatto and Grasselli (2013), and Ahlip et al. (2017).

The paper is structured as follows. Section 2 describes the market conventions for quoting option prices. Section 3 addresses the Arbitrage-Free Approach and its identification from market data. Section 4 reviews the Vanna Volga Approach. Section 5 benchmarks the Arbitrage-Free Approach to the Vanna Volga Approach for both empirical scenarios considered. Furthermore, it discusses the comparative statics, the parameter estimates, and admissible parameterizations. Section 6 concludes.

## FX market conventions

This section defines the market and pricing conventions on FX option markets. Details can also be found in, for example, Reiswich and Wystup (2010, 2012), or the textbooks of Clark (2011) and Wystup (2017). Section 2.1 addresses implied volatilities and deltas. Section 2.2 reviews at-the-money (ATM) conventions, risk reversals, and butterflies, which are quoted on the market.

### Implied volatility and deltas

Standard market practice is to communicate FX option prices in terms of implied volatilities assuming the Garman and Kohlhagen (1983) model. Consider the forward exchange rate $F_t^T$ with delivery at *T* in FORDOM quotation (i.e., the rate defines the amount of domestic currency that is exchanged for one unit of foreign currency) at time *t*. According to a standard argument it is related to the spot exchange rate $S_t$ through the relationship

1 $$\begin{aligned} F_t^T= & {} \frac{S_t B_f(t,T)}{B_d(t,T)}, \end{aligned}$$

where $B_f(t,T)$ and $B_d(t,T)$ are the prices of a foreign and a domestic zero bond at time *t* each paying off one unit of foreign and domestic currency at *T*. Hence, under the forward measure, it may be assumed that

2 $$\begin{aligned} \frac{dF^T_t}{F^T_t}= & {} \sqrt{v_t^T} dW^T_t, \end{aligned}$$

where $v_t^T$ is the instantaneous variance of the forward exchange change. It may be time and state dependent. The Wiener process $W^T_t$ is the risk factor that drives the forward rate with delivery at *T*. If the interest rates and the variance of the exchange rate are constant then the Garman and Kohlhagen (1983) option pricing formula applies. The price $V_t^T(X)$ of a European option with strike price *X* and expiry *T* in domestic currency is

3 $$\begin{aligned} V_t^T(X)=c B_d(t,T)\left[ F_t^T \Phi (c d_1)-X \Phi (c d_2)\right] \end{aligned}$$

where

$$\begin{aligned} d_1= & {} \frac{\ln \left( \frac{F_t^T}{X}\right) +\frac{1}{2}{\bar{\sigma }}^2 (T-t)}{{\bar{\sigma }} \sqrt{T-t}}\\ d_2= & {} d_1 - {\bar{\sigma }} \sqrt{T-t}, \end{aligned}$$

$c=1$ ($c=-1$) for a call (put option), and $\Phi (\cdot )$ is the cumulative distribution function of the standard normal probability distribution.^2^ The constant ${\bar{\sigma }}=\sqrt{v^T_t}$ is referred to as the (implied) volatility of the option. It should be stressed that this does not imply that market participants “believe” in the Garman/Kohlhagen model, that is, in log-normally distributed forward exchange rates. Instead, the model is used as a communication device for prices. In fact, the presence of volatility smiles is at odds with this assumption.

FX option markets also differ in another important aspect from other option markets such as stock options or interest rate options since implied volatilities are quoted with respect to delta and not strike. In practice, simple and premium adjusted deltas have to be distinguished. For each type, spot and forward deltas exist. The focus of this paper are simple (non-premium adjusted) deltas.^3^ The spot delta of an option $\Delta _S$ is defined as

4 $$\begin{aligned} \Delta _S=c B_f(t,T)\Phi (c d_1). \end{aligned}$$

Intuitively, the spot delta gives the amount of foreign currency that must be purchased to replicate the option in the Garman/Kohlhagen setup. It includes the foreign discount factor that applies to the expiry.

The simple (non-premium adjusted) forward delta of an option $\Delta _F$ is given by

5 $$\begin{aligned} \Delta _F=c \Phi (c d_1)=\frac{\Delta _S}{B_f(t,T)}. \end{aligned}$$

This delta assumes that hedging is carried out using forward contracts. Note that the foreign discount factor disappears from the definition. In practice, forward deltas are frequently used to price long-term options while short-term option volatilities are quoted with reference to spot deltas. Non-premium adjusted deltas are usually applied when option prices are quoted in terms of domestic currencies. This is, for example, the case for EURUSD options discussed in greater detail below in Sect. 5. These options are written on the EUR as foreign currency. Option prices are paid in USD, that is, domestic currency.

### At-the-money volatilities, risk reversals, and butterflies

In practice, option prices are communicated through at-the-money (ATM) volatilities as well as the prices of risk reversals and butterflies. Concerning the ATM volatilities several market conventions exist. In this paper, we focus on the ATM-delta neutral straddel convention (ATM-DNS).^4^ The ATM-DNS quotation assumes a delta neutral straddle, that is, a long position in a call and a put option with identical strike that is delta neutral on aggregate. For non-premium adjusted delta, this implies that

6 $$\begin{aligned} \Phi (d_1)=\Phi (-d_1)=\frac{1}{2} \end{aligned}$$

for both forward and spot delta conventions. Consequently, the ATM-strike $X_{ATM}$ for a given ATM-volatility $\sigma _{ATM}$ is

$$\begin{aligned} X_{ATM}=F(t,T) e^{\frac{1}{2} \sigma _{ATM}^2 (T-t)}. \end{aligned}$$

FX volatility smiles are identified from quotes for *risk reversals* (RR) and *butterflies* (BF). A ${\bar{\Delta }}$-risk reversal is a portfolio consisting of a long call with delta $\Delta _{S,F}^C={\bar{\Delta }}$ and a short put with $\Delta _{S,F}^P=-{\bar{\Delta }}$. Per market convention the price of the risk reversal $RR_{{\bar{\Delta }}}$ is quoted as

7 $$\begin{aligned} RR_{{\bar{\Delta }}} = \sigma ^C\left( {\bar{\Delta }}\right) -\sigma ^P\left( -{\bar{\Delta }}\right) , \end{aligned}$$

where $\sigma ^C\left( {\bar{\Delta }}\right)$ and $\sigma ^P\left( -{\bar{\Delta }}\right)$ are the implied volatilities of the call and the put option. Intuitively, risk reversal quotes measure the skewness of the volatility smile.

The market quote of a butterfly refers to a delta neutral strangle, that is, a long position in a call and a put option. “Smile” and “broker” strangles have to be distinguished. In a smile strangle the options underlying a ${\bar{\Delta }}$-smile strangle are the same as those underlying a ${\bar{\Delta }}$-risk reversal while they are different in a broker strangle. The focus in this paper is on smile strangles. In the following, the notion of a butterfly will always refer to this type of contract. For a smile strangle, the ${\bar{\Delta }}$-butterfly is quoted as

8 $$\begin{aligned} BF_{{\bar{\Delta }}} =\frac{1}{2} \left[ \sigma ^C\left( {\bar{\Delta }}\right) +\sigma ^P\left( -{\bar{\Delta }}\right) \right] -\sigma _{ATM}, \end{aligned}$$

where $\sigma _{ATM}$ is the at-the-money volatility. A butterfly measures the steepness of the smile. Its quote is equal to the average volatility minus the ATM-volatility.

## Arbitrage-free approach

This section addresses the Arbitrage-Free Approach for FX smile construction. The approach is nested in the general model for near-term implied volatility dynamics that is proposed by Carr and Wu (2016). The section is organized as follows: Sect. 3.1 addresses the model of near-term dynamics and derives the no-arbitrage condition with respect to delta as well as the symmetry property. Section 3.2 discusses the model identification, admissible parameterizations, and closed-form solutions for arbitrage-free implied volatilities. The focus is on non-premium adjusted deltas.^5^

### Near-term dynamics and symmetry property

Consider the general dynamics of the forward exchange rate in Eq. (2). Carr and Wu (2016) assume that the implied volatility $\sigma _t(X)$ of an option with strike *X* and delivery *T* follows the near-term diffusive dynamics

$$\begin{aligned} d\sigma _t(X)=\mu _t^T dt + \omega _t^T dZ_t, \end{aligned}$$

where $\mu _t^T$ and $\omega _t^T$ are time and state dependent. The implied volatility $\sigma _t(X)$ replaces the constant ${\bar{\sigma }}$ in Eq. (3). Moreover, $Z_t$ is a standard Wiener process under the given forward measure. This factor drives implied volatilities of the entire volatility surface (i.e., all strikes and expiry dates). Subsequently, the paper refers to $\mu ^T_t$ and $\omega _t^T$ as the near-term expected return and the near-term volatility of implied volatility, respectively. Given that forward exchange rates might be exposed to different risk factors the near-term correlation $\rho ^T_t={\mathbb {E}}\left[ dW^T_t dZ_t\right]$ between implied volatility and the forward rate depends on the delivery date. Similarly, the parameters $\mu ^T_t$, $w^T_t$, and $\rho ^T_t$ can be functions of delivery date but not of strike. To lighten the notation, their dependency on *T* as well as the dependency on implied volatility on the strike are frequently dropped in the following.

As discussed in Carr and Wu (2016), the implied volatility of a put option meets the no-arbitrage constraint

9 $$\begin{aligned} \frac{1}{2} \sigma _t^2-\mu _t\sigma _t\tau -\frac{1}{2}v_t-\rho _t\frac{\omega _t}{\sigma _t}\sqrt{v_t}\left( \log \frac{X}{F_t}+\frac{\sigma _t^2\tau }{2}\right) -\frac{1}{2}\frac{\omega _t^2}{\sigma _t^2}\left[ \left( \log \frac{X}{F_t}\right) ^2-\frac{1}{4}\sigma _t^4\tau ^2\right] =0, \end{aligned}$$

where $\tau =T-t$. This no-arbitrage constraint holds for general diffusive dynamics of the implied volatility. Carr and Wu (2011, 2016) discuss various specifications for these dynamics. In this paper, implied volatility dynamics similar to their “Proportional Volatility Dynamics”^6^ are considered. In this special case, $\mu _t=\theta _t^T \sigma _t$ and $\omega _t^T=\xi _t^T \sigma _t$ such that the implied volatility dynamics are given by

10 $$\begin{aligned} \frac{d\sigma _t}{\sigma _t}=\theta _t^T dt + \xi _t^T dZ_t. \end{aligned}$$

In Carr and Wu (2016) the drift and the volatility parameters of the implied volatility process are exponentially decreasing with respect to the options residual life, that is, $\theta _t^T=m_t e^{-\eta _t \tau }$ and $\xi _t^T=w_t e^{-\eta _t \tau }$. If $\eta _t>0$ then implied volatilities are negatively related to $\tau$. Carr and Wu (2016) motivate this choice with the empirical observation that implied volatilities of options with expiries in the more distant future are less risky. Consequently, the entire volatility surface can be described parsimoniously with a few parameters only. The focus of this paper is on volatility smiles and in particular on inter- and extrapolation of implied volatilities, though. Therefore, this structure is not imposed on the drift and volatility parameters. Again, to lighten notation the dependency on *T* of the the drift and volatility parameters will be dropped in the following.

The no-arbitrage condition stated in Eq. (9) depends on the forward rate and the strike. On FX option markets, implied volatilities are quoted in terms of delta. As an alternative, consider the moneyness variable *d* to be defined as

11 $$\begin{aligned} d=\Phi ^{-1}\left( -\frac{\Delta ^P_S}{B_f(t,T)}\right) =\Phi ^{-1}\left( -\Delta _F^P\right) . \end{aligned}$$

This variable can be calculated from put option deltas $\Delta _{S,F}^P$ that are observable on the market. Furthermore, consider the change of variables

12 $$\begin{aligned} \ln \left( \frac{X}{F}\right) =\sigma _t \sqrt{\tau }d+\frac{1}{2}\sigma _t^2\tau . \end{aligned}$$

Consequently, for implied volatilities given in Eq. (10) and the change of variables in Eq. (12) the no-arbitrage constraint in Eq. (9) implies that arbitrage-free implied volatilities are given as the roots of the cubic polynomial $g_d(\sigma )$, which is defined as

13 $$\begin{aligned} g_d(\sigma _t)= d \xi _t ^2 \tau ^{3/2} \sigma _t^3+\left[ \xi _t \tau \left( d^2 \xi _t +2 \rho _t \sqrt{v_t}\right) +2 \theta _t \tau -1\right] \sigma _t^2+2 d \xi _t \rho _t \sqrt{v_t}\sqrt{\tau } \sigma _t+v_t. \end{aligned}$$

The following observations are worth mentioning: First, the no-arbitrage condition is independent of forward rates and strikes. Instead it relates arbitrage-free implied volatilities through the moneyness variable to market deltas directly. In particular, FX smile construction does not require data on forward exchange rates potentially introducing statistical noise. Second, Carr and Wu (2016) write the no-arbitrage conditions for their Proportional Volatility Dynamics in terms of the moneyness variable $k=\log \left( \frac{X}{F_t}\right)$, which is applicable for stock options. They obtain the implied variance as the roots of a (simpler) quadratic polynomial.^7^ Eq. (13) highlights that if the moneyness variable *d* is considered then implied volatilities are given by the roots of a cubic polynomial in fact. Third, Carr and Wu (2011) also consider a “Square-Root Variance Model” (SRV), which identifies implied volatilities as the roots of a quadratic polynomial and with respect to the moneyness variable $z=d+\sigma _t\sqrt{\tau }$.^8^ However, a computational advantage of the cubic polynomial in Eq. (13) is that it has a symmetry property, which is not present in the SRV model. This symmetry property states that

14 $$\begin{aligned} g_d(\sigma _t)=g_{-d}(-\sigma _t). \end{aligned}$$

Economically, only positive implied volatilities and thus the positive roots of the cubic polynomial (13) are meaningful. By the symmetry property, negative roots also have an economic interpretation. Assume that $X_P$ and $X_C$ are the strikes of the put and the call option of the ${\bar{\Delta }}$-risk reversal/butterfly. Let *d* be the moneyness variable of a put option with strike $X_P$ and delta $-{\bar{\Delta }}_{S,F}$ according to Eq. (11). Then the forward and spot deltas of the put option with strike $X_C$ are given by

$$\begin{aligned} {\bar{\Delta }}^P_{F}\left( X_C\right) =\frac{{\bar{\Delta }}^P_{S}\left( X_C\right) }{B_f(t,T)}=-\Phi (-d). \end{aligned}$$

Hence, arbitrage-free implied volatilities are given by the positive roots of $g_{-d}$. The symmetry property states that the positive roots of $g_{-d}$ are the negative roots of $g_{d}$. Therefore, in a nutshell, the quotes of the ${\bar{\Delta }}$-risk reversal/butterfly are both determined by the roots of $g_d$. This cubic polynomial can have up to three real roots.

### Model identification

FX volatility smiles are usually characterized by communicating a few benchmark quotes including ATM-volatilities as well as quotes for risk reversals and butterflies. Other implied volatilities have to be inter- and extrapolated. The application of the Arbitrage-Free Approach requires the latent parameters $\theta _t$, $\rho _t$, and $\xi _t$ that affect near-term implied volatility dynamics. These parameters have to be chosen such that the roots of the cubic polynomial in Eq. (13) yield implied volatilities for the option contracts that drive benchmark quotes and that can be observed on the market.

To begin with ATM-volatility, note that the determination of the roots of the cubic polynomial simplifies when the moneyness variable $d=0$. Per Eq. (6) this is, for example, the case for the ATM-put option according to the ATM-DNS convention. The cubic polynomial defined in Eq. (13) then becomes

$$\begin{aligned} g_0(\sigma _t)= \left[ 2\xi _t\rho _t \tau \sqrt{v_t} +2 \theta _t \tau -1\right] \sigma _t^2+v_t. \end{aligned}$$

The ATM-volatility $\sigma _{ATM}$ thus meets the no-arbitrage condition

15 $$\begin{aligned} \sigma _{ATM}=\sqrt{\frac{v_t}{1-2\xi _t\rho _t \tau \sqrt{v_t} -2 \theta _t \tau }}. \end{aligned}$$

In the following the forward rate variance is identified as $v=\sigma _{ATM}^2$. An advantage of this identification is that the latent forward rate variance is linked to a quantity, which is quoted directly on the market. Note that as the expiry $\tau$ approaches zero, Eq. (13) implies that the FX volatility smile is flat with respect to *d* and its level is equal to the volatility of the forward rate, which approaches the spot rate.^9^ As a consequence of this identification, Eq. (15) yields

16 $$\begin{aligned} \theta _t=-\rho _t \xi _t\sqrt{v_t}=-\rho \xi _t\sigma _{ATM}. \end{aligned}$$

Thus, the near-term expected return of implied volatility $\theta _t$ follows directly from the covariance of the forward exchange rate and implied volatilities. Eq. (16) ensures that the observed ATM-volatility is matched by construction.

The parameters $\rho _t$ and $\xi _t$ remain to be calibrated to benchmark quotes, that is, the implied volatilities of the two standard options underlying risk reversals and butterflies. From the symmetry property, it follows that the quotes of a $\Delta$-risk reversal and a $\Delta$-butterfly are identified by the roots of a single cubic polynomial. Therefore, it is sufficient to vary $\rho _t$ and $\xi _t$ such that the roots of the cubic polynomial concur with the prices of the benchmark contracts (risk reversal/butterfly) while the choice of $\theta _t$ is constrained according to Eq. (16). The symmetry property (14) implies that to ensure that quotes of risk reversals and butterflies exist, restrictions apply. The cubic polynomial in Eq. (13) must have at least one positive and one negative root. A necessary condition is that more than one real root exists. From a standard argument three real roots do exist if the discriminant $D<0$, where *D* is defined as

17 $$\begin{aligned} D= & {} \left( \frac{q}{2}\right) ^2+\left( \frac{p}{3}\right) ^3 \nonumber \\ p= & {} \frac{3 \alpha \gamma -\beta ^2}{3\alpha ^2} \nonumber \\ q= & {} \frac{2\beta ^3-9\alpha \beta \gamma +27\alpha ^2\delta }{27\alpha ^3}, \end{aligned}$$

and

$$\begin{aligned} \alpha= & {} d \xi _t ^2 \tau ^{3/2} \\ \beta= & {} \xi _t \tau \left( d^2 \xi _t +2 \rho _t \sqrt{v_t}\right) +2 \theta _t \tau -1 \\ \gamma= & {} 2 d \xi _t \rho _t \sqrt{v_t}\sqrt{\tau } \\ \delta= & {} v_t \end{aligned}$$

are the coefficients of the cubic polynomial defined in Eq. (13).^10^ When parameters meet this admissibility constraint then the three roots are given by the well-known solution

18 $$\begin{aligned} \sigma _{0,1}= & {} \frac{{\mathcal {A}}}{3 \root 3 \of {2} \alpha }-\frac{\root 3 \of {2} \left( 3 \alpha \gamma -\beta ^2\right) }{3 \alpha {\mathcal {A}}}-{\mathcal {B}} \nonumber \\ \sigma _{0,2}= & {} -\frac{\left( 1-i \sqrt{3}\right) {\mathcal {A}}}{6 \root 3 \of {2} \alpha }+\frac{\left( 1+i \sqrt{3}\right) \left( 3 \alpha \gamma -\beta ^2\right) }{3\rtimes 2^{2/3} \alpha {\mathcal {A}}}-{\mathcal {B}} \nonumber \\ \sigma _{0,3}= & {} -\frac{\left( 1+i \sqrt{3}\right) {\mathcal {A}}}{6 \root 3 \of {2} \alpha }+\frac{\left( 1-i \sqrt{3}\right) \left( 3 \alpha \gamma -\beta ^2\right) }{3\rtimes 2^{2/3} \alpha {\mathcal {A}}}-{\mathcal {B}} \end{aligned}$$

with

$$\begin{aligned} {\mathcal {A}}= & {} \root 3 \of {\sqrt{\left( 27 \alpha ^2 \delta -9 \alpha \beta \gamma +2 \beta ^3\right) ^2-4 \left( \beta ^2-3 \alpha \gamma \right) ^3}-27 \alpha ^2 \delta +9 \alpha \beta \gamma -2 \beta ^3} \\ {\mathcal {B}}= & {} \frac{\beta }{3 \alpha } \end{aligned}$$

and $i^2=-1$. The benchmark quotes for the risk reversal and the butterfly then follow from a positive and a negative root of the cubic polynomial. Assume that $\theta _t$ is chosen according to Eq. (16). Appendix A discusses that there is always at least one positive and one negative root when the discriminant $D<0$ and $\rho _t < 0$.^11^ If $\rho _t > 0$ then a necessary condition for the existence of at least one positive and at least one negative root is that $\xi _t^2<\frac{1}{d^2 \tau }$. Thus, the volatility parameter $\xi _t$ must not be too large. Note that the maximum value of $\xi _t$ depends on the moneyness variable *d*. The maximum value decreases when moving from the ATM volatility to the wings of the FX volatility smile.

To identify the prices of the risk reversal and the butterfly (and, thus, the FX smile as a whole) only two roots of the cubic polynomial are required. However, if $D<0$ then three (different) real roots exist. There is one degree of freedom concerning the choice of roots. In the following, the smallest negative and the largest positive roots are considered. In numerical applications the absolute value of the third root is usually very high and clearly outside the range of empirically observed FX implied volatilities. Other criteria are also possible. For example, the choice of the roots could be based on the symmetry of the FX volatility smile: The lower (larger) the difference between the absolute values of the positive and the negative roots considered the larger (lower) is the difference between the implied volatilities of the put and the call options of a risk reversal/butterfly and thus the more (less) symmetrical is the resulting FX volatility smile.^12^

## Vanna volga approach

As a benchmark, the Vanna Volga Approach is considered.^13^ This is a standard approach in practice. It assumes that the Garman/Kohlhagen model applies, that is, the volatility smile is flat and all options have the same model implied volatility $\sigma ^{GK}$ with option price $C^{GK}$. A hedging portfolio is constructed, which immunizes an option with respect to vega, vanna, and volga exposures, that is, model risk. Vega is the sensitivity of an option price with respect implied volatility changes ($C^{GK}_{\sigma }$). Vanna gives the sensitivity of vega with respect to changes in underlying forward exchange rate ($C^{GK}_{\sigma F}$). Volga measures the sensitivity of vega with respect to implied volatility changes ($C^{GK}_{\sigma \sigma }$).

To match vega, vanna, and volga exposures, three benchmark options with strikes $K_1< K_2 < K_3$ are required. Their observable market prices and implied volatilities are $C^M(K_1)$, $C^M(K_2)$, and $C^M(K_3)$ as well as $\sigma _1$, $\sigma _2$, and $\sigma _3$, respectively. In the presence of a volatility smile, these prices and implied volatilities are not in line with the Garman/Kohlhagen model prices $C^{GK}(K_i)$, $i \in \{1,2,3\}$ and the Garman/Kohlhagen implied volatility $\sigma ^{GK}$. In the following, $K_2$ is the strike of an ATM-option. The strikes $K_1$ and $K_3$ are the strikes of options that constitute a $\Delta$-risk reversal/butterfly.

Let $x_i$, $i\in \{1,2,3\}$, be the necessary positions in options with strikes $K_i$, $i\in \{1,2,3\}$, to hedge an option with strike *K* against vega, vanna, and volga risk. The Vanna Volga price of an option $C^{VV}(K)$ is given by

19 $$\begin{aligned} C^{VV}(K)= & {} C(K)^{GK}+\sum _{i=1}^3 x_i\left[ C^M(K_i)-C^{GK}(K_i)\right] \end{aligned}$$

with

$$\begin{aligned} C_{\sigma }^{GK}(K)= & {} \sum _{i=1}^3 x_i C_{\sigma }^{GK}(K_i)\\ C_{\sigma \sigma }^{GK}(K)= & {} \sum _{i=1}^3 x_i C_{\sigma \sigma }^{GK}(K_i)\\ C_{\sigma F}^{GK}(K)= & {} \sum _{i=1}^3 x_i C_{\sigma F}^{GK}(K_i) \end{aligned}$$

Intuitively, the Vanna Volga price $C^{VV}(K)$ is the Garman/Kohlhagen model price $C^{GK}(K)$ plus the hedging cost for vega, vanna, and volga risk. It is important to realize that the Vanna Volga and the Garman/Kohlhagen model prices deviate from each other since model risks are hedged using the observed market prices $C^M(K_i)$, $i \in \{1,2,3\}$ (and not the Garman/Kohlhagen prices $C^{GK}(K_i)$).

The Vanna Volga implied volatility $\sigma ^{VV}(K)$ of an option with strike *K* can then be approximated as^14^

20 $$\begin{aligned} \sigma ^{VV}(K)\approx \sigma _2+\frac{-\sigma _2+\sqrt{\sigma _2^2+d_1(K)d_2(K)\left( 2\sigma _2D_1(K)+D_2(K)\right) }}{d_1(K)d_2(K)}, \end{aligned}$$

where

$$\begin{aligned} D_1= & {} \eta _1(K)-\sigma _2\\ D_2= & {} \frac{ln\frac{K_2}{K}ln\frac{K_3}{K}}{ln\frac{K_2}{K_1}ln\frac{K_3}{K_1}} d_1(K_1)d_2(K_1)\left( \sigma _1-\sigma _2\right) ^2 +\frac{ln\frac{K}{K_1}ln\frac{K}{K_2}}{ln\frac{K_3}{K_1}ln\frac{K_3}{K_2}} d_1(K_3)d_2(K_3)\left( \sigma _3-\sigma _2\right) ^2 \\ \eta (K)= & {} \frac{ln\frac{K_2}{K}ln\frac{K_3}{K}}{ln\frac{K_2}{K_1}ln\frac{K_3}{K_1}} \sigma _1 + \frac{ln\frac{K}{K_1}ln\frac{K_3}{K}}{ln\frac{K_2}{K_1}ln\frac{K_3}{K_2}} \sigma _2+ \frac{ln\frac{K}{K_1}ln\frac{K}{K_2}}{ln\frac{K_3}{K_1}ln\frac{K_3}{K_2}}\sigma _3 \end{aligned}$$

The functions $d_1$ and $d_2$ are given in Eq. (3). They are evaluated at $\sigma ^{GK}=\sigma _2$. This approximation is based on a Taylor expansion at second order. As observed by Castagna (2010) it is “extremely accurate”. Note that market implied volatilities $\sigma _1$, $\sigma _2$, and $\sigma _3$ are matched by construction, that is, $C^M(K_i)=C^{VV}(K_i)$, $i\in \{1,2,3\}$.

Similar to the Arbitrage-Free Approach, the Vanna Volga Approach constructs the entire smile from three benchmark options only. Even though implied volatilities are determined with respect to strikes, Eq. (20) implies that implied volatilities are independent of the forward rates when they are mapped with respect to delta. Similar to the Arbitrage-Free Approach, forward rates are not required to compute the smile.^15^ Beyond that and more importantly, the Vanna Volga Approach is not a coherent financial model and arbitrage-free option prices/implied volatilities are not guaranteed. Rather, it is an inter- and extrapolation approach.

## Application to market data

This section summarizes the results of an empirical application of the model to market prices during on a “normal” trading day and during a stress scenario. Section 5.1 provides details on the data. Section 5.2 compares interpolation and extrapolation in the Arbitrage-Free Approach and the Vanna Volga Approach. Section 5.3 discusses comparative statics and presents parameter estimates in the Arbitrage-Free Approach. Section 5.4 addresses admissible parameters.

### Data

Similar to Ammann and Feser (2019) an exemplary crisis and a normal scenario are distinguished. Therefore, two observation days are considered. The first observation day (April 01, 2020, “volatile day”) is during a period of market stress (Coronavirus crisis). This is also indicated by the VIX, which was about 57 on that day. The second day (October 09, 2019, “normal day”) is about six months earlier when markets were less volatile with a VIX of about 19. For these days, benchmark quotes for Euro (EURUSD) currency options are considered. These are the ATM-volatilities (ATM-DNS convention), the $\Delta 25$-risk reversals (RR), and the $\Delta 25$-butterflies (BF). Mid-quotes for the 1-month, 2-month, 3-month, 6-month, 9-month, 1-year, 2-year, 3-year, and 5-year expiries are obtained from Refinitive Eikon.^16^ Quotes for short expiries up to one year are given in terms of spot deltas. Spot deltas are linked to EUR-discount factors through Eq. (4). Therefore, EUR-discount factors are calculated from mid-deposit rates for these maturities.^17^

**Table 1:** Summary Statistics: Spreads

	April 01, 2020				Oct. 09, 2019			
	Median	Min	Max	StdDev	Median	Min	Max	StdDev
*A: Mid-Quotes*								
ATM	0.08925	0.08500	0.09575	0.00331	0.05750	0.05113	0.07475	0.00759
$\Delta$25-RR	− 0.00875	− 0.01100	− 0.00550	0.00174	0.00000	− 0.00075	0.00100	0.00050
$\Delta$25-BF	0.00588	0.00513	0.00625	0.00040	0.00240	0.00130	0.00275	0.00044
*B: Spreads*								
ATM	0.00800	0.00800	0.01000	0.00067	0.00200	0.00150	0.00250	0.00041
$\Delta$25-RR	0.00800	0.00800	0.01200	0.00147	0.00350	0.00200	0.00750	0.00176
$\Delta$25-BF	0.00250	0.00149	0.00650	0.00169	0.00200	0.00150	0.00450	0.00114

The table shows the statistics across expiry dates of spreads (ask minus bid, Panel A) and mid-quotes (average of bid and ask, Panel B) for ATM volatilities and $\Delta$25 risk reversal/butterfly on the Euro on both observation days. The expiry dates considered are 1 month, 2 months, 3 months, 6 months, 9 months, 1 year, 2 years, 3 years, and 5 years. Data source is Refinitiv Eikon, RICs: EURXXO, EURXXRR, and EURXXBF, where XX is the expiry.(e.g., for the 1 month and 1 year expiries XX is 1M and 1Y, respectively.)

Table 1 provides summary statistics of mid-market quotes (Panel A) and bid-ask spreads (Panel B) of all available expiries for each contract type. The reported statistics are medians, min-max ranges, and volatilities of all available expiries (cross sectional data) on both observation dates. In line with intuition, median ATM-volatilities are higher on the volatile day (about 9% compared to about 6%). Moreover, the median $\Delta 25$-risk reversal quote is negative on the volatile day indicating that $\Delta 25$-put options are more expensive than $\Delta 25$-call options. In comparison, on the normal day the median quote for $\Delta 25$-risk reversal is around zero. Smiles are also steeper on the volatile day. The median $\Delta 25$-butterfly is higher than on the normal day. Median spreads are larger for all products on April 01, 2020 suggesting a greater reluctance of market participants to enter into option positions in the presence of market stress.

Per Eqs. (7) and (8), implied volatilities for the $\Delta 25$-call ($\sigma ^C_{25}$) and put ($\sigma ^P_{25}$) follow from the quotes of the $\Delta 25$-risk reversals $RR_{25}$, the $\Delta 25$-butterfly $BF_{25}$ and the ATM-volatility $\sigma _{ATM}$ as

21 $$\begin{aligned} \sigma ^C_{25}= & {} \sigma _{ATM}+\frac{1}{2}RR_{25}+BF_{25} \nonumber \\ \sigma ^P_{25}= & {} \sigma _{ATM}-\frac{1}{2}RR_{25}+BF_{25}. \end{aligned}$$

In total, three benchmark implied volatilities are available for each expiry ($\sigma ^P_{25}$, $\sigma _{ATM}$, and $\sigma ^C_{25}$). Since the model requires put deltas as inputs, all call deltas are transformed into put deltas.

Note that the given butterflies are the prices of broker strangles. Unlike the smile strangle the strikes of the call and the put option underlying the $\Delta 25$-strangle are different from the option prices from which the $\Delta 25$-risk reversal is constructed. Therefore, Eq. (21) approximates the true implied volatilities assuming that the quotes for smile and market strangles are similar. As discussed in Reiswich and Wystup (2012), the error should be small, though, given that the smile is not highly skewed.^18^

### Smile inter- and extrapolation

In the Arbitrage-Free Approach, ATM-volatilities can be matched according to Eq. (16). The remaining two parameters $\xi _t$ and $\rho _t$ are calibrated by considering the options underlying the $\Delta 25$-benchmark contracts. As noted earlier, their implied volatilities are given by the smallest positive and the largest negative root of a single cubic polynomial. This cubic polynomial, thus, defines level, steepness, and skewness of the smile. Similarly, the benchmark Vanna Volga Approach also requires only three option prices to define the smile according to Eq. (20).

**Fig. 1:**
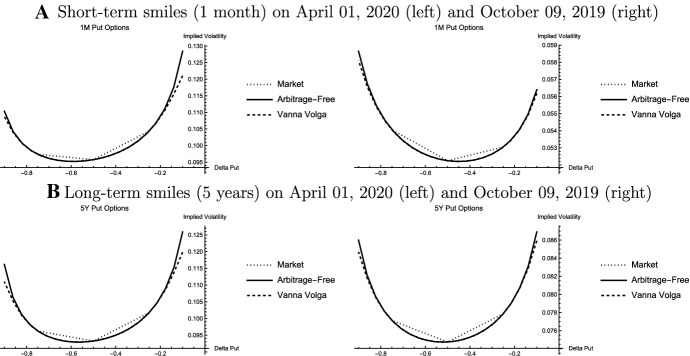
Model and Market Smiles. The figure shows implied volatilities of the three benchmark contracts (dotted lines) in comparison to the smiles according to Arbitrage-Free Approach (“Arbitrage-Free”, solid lines) and the Vanna Volga Approach (“Vanna Volga”, dashed lines) as a function of $\Phi (d)$ (“Delta Put”, d: moneyness variable). The smiles on the left and the right refer to April 01, 2020 and October 09, 2019, respectively

Figure 1 compares the implied volatilities of the implied volatilities of the ATM- and the $\Delta 25$-options to the smiles from the Arbitrage-Free Approach and the Vanna Volga Approach. Results are shown exemplary for short-term 1-month and long-term 5-year options. They are similar for the other expiries. Per construction both smile approaches match ATM-volatilities. The Vanna Volga Approach also fits implied volatilities of $\Delta 25$-options while there is a small, though negligible, numerical error for Arbitrage-Free Approach. Concerning interpolation of implied volatilities for deltas between those of the option underlying the $\Delta 25$-benchmark contracts (risk reversal and butterfly), both approaches yield very similar results. Differences are observed when the smile is extrapolated to the wings. In particular on the volatile day, implied volatilities from the Vanna Volga Approach are significantly lower than those from the Arbitrage-Free Approach. Economically, this observation may be important, for example, for the estimation of crash indices like the Jump and Tail Index (JTIX) or the Rare Disaster Concern Index (RIX) suggested by Du and Kapadia (2012) and Gao et al. (2018), respectively. On the normal trading day, differences in the wings are also observed for both approaches. However, these differences are less pronounced than on the volatile day.

### Comparative statics and estimated parameters

**Fig. 2:**
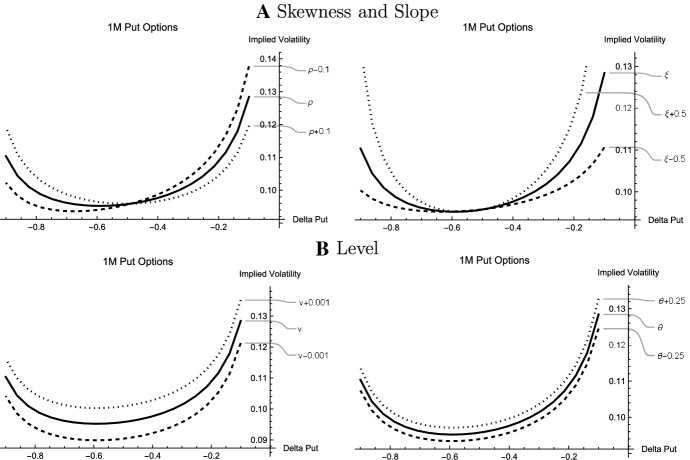
Comparative Statics: Arbitrage-Free Approach. The figure shows comparative statics for the base case parameters estimated for 1 month EUR options on April 01, 2020 (Coronavirus Crisis): $\rho _t=-0.1121$, $\xi _t=1.6654$, $v_t=0.0092$, and $\theta _t=-\sqrt{v_t} \xi _t \rho _t= 0.0179$. The near-term correlation ($\rho _t$), near-term volatility of implied volatility ($\xi _t$), the variance of the forward rate ($v_t$), and the near-term expected return of implied volatility ($\theta _t$) are varied by $\pm 0.1$, $\pm 0.5$, $\pm 0.001$, and $\pm 0.25$, respectively. Each plot shows the sensitivity of the smile with respect to a variation in one parameter while the other parameters are equal to the base case. Solid (dotted, dashed) smiles represent base cases (cases with increased parameters, cases with decreased parameters)

Figure 2 has the comparative statics. As base case, the estimated parameters on the volatile day (April 01, 2020) are considered.^19^ The relationships are demonstrated for 1-month options. Panel A shows the parameters that drive the skewness and the slope of the smile. These are the correlation coefficient $\rho _t$ and the volatility parameter $\xi _t$. Increasing the correlation reduces implied volatilities with a small put delta (in absolute terms), that is, implied volatilities for low strikes. Conversely, put options with high absolute deltas (high strikes) have higher implied volatilities and thus prices. The opposite effect is observed when the correlation decreases. Thus, $\rho _t$ can be viewed as a “skewness parameter”. In contrast, the volatility $\xi _t$ is a “steepness parameter”. The relationship between steepness and the choice of $\xi _t$ is positive while it has very little impact on skewness. The impact on the level of both parameters is very small.

Panel B of Fig. 2 addresses the parameters driving the level of the smile. These are the parameters $\theta _t$ and $v_t$. The impact of both quantities on the volatility smile is similar. There is a positive relationship between the level and $\theta _t$ as well as $v_t$ while skewness and steepness are only mildly affected. This also motivates numerically the identification of $\theta _t$ from the covariance of forward exchange rate and implied volatility rather than using it as “free parameter” (Eq. (16)).

**Fig. 3:**
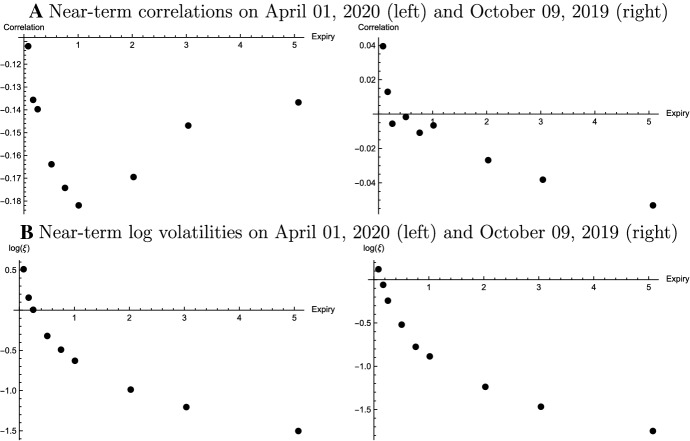
Calibrated Model Parameters: Arbitrage-Free Approach. The figure shows near-term volatilities of implied volatilities ($\log \xi _t^T$) and near-term correlations ($\rho _t^T$) with respect to option expiry dates. The figures on the left and the right refer to April 01, 2020 and October 09, 2019, respectively

Empirically, Fig. 3 depicts the estimation results for the near-term volatility of implied volatility $\xi _t$ and the near-term correlation $\rho _t$ for the expiries considered. Panel A shows the estimates for the correlation parameter $\rho _t$, that is, the skewness parameter. For both observation dates, the estimates of $\rho _t$ depend on the expiry of the smile. On the volatile day (April 01, 2020), correlations are negative throughout and a humped shaped function of expiry. Again, this coincides with the mid-quotes for risk reversals, which are negative for all expiries and have a minimum for options with expiry in one year. On the normal trading day (October 09, 2019), the relationship between the correlation and the expiry is negative. For short-term smiles, the correlation is positive while the opposite is true for long-term smiles. The same is true for the mid-quotes of the $\Delta 25$- risk reversals. These quotes are positive or zero for expiries up to one year and negative beyond.

Panel B shows that the relationship between the log of the estimated volatility of implied volatility $\xi _t$ and the expiration of the smile is negative. As discussed, the volatility of implied volatility can be seen as the steepness parameter. The estimates demonstrate that the observed steepness of short-term smiles implies high values for $\xi _t$. The exponential rate of decay is higher for short-term than for long-term expiries. This contrasts the “Proportional Volatility Dynamics” specification in Carr and Wu (2016) that assumes a constant exponential rate of decay of both $\theta _t$ and $\xi _t$. Comparing both observation dates, the estimates for $\xi _t$ are higher on April 01, 2020. This is a consequence of the increased steepness of the smiles during the more volatile trading period.

### Admissible parameterizations

To explore the set of admissible parameterizations of arbitrage-free smiles, the maximum admissible near-term volatility $\xi _t^{max}$ is computed given the other calibrated parameters ($v_t$, $\theta _t$, and $\rho _t$) on both trading days (April 01, 2020 and October 09, 2019). The discriminant defined in Eq. (17) must be smaller than zero to ensure that the cubic polynomial in Eq. (13) has three real roots such that a risk reversal and a smile spread can be priced. Furthermore, $\xi _t^{max}< \frac{1}{d^2 \tau }$ if $\rho _t>0$ to ensure that there is at least one positive and one negative real root. The boundary $\xi _t^{max}$ is determined for forward put (call) deltas of −0.55 (0.45), −0.75 (0.25), and −0.95 (0.05).^20^

**Fig. 4:**
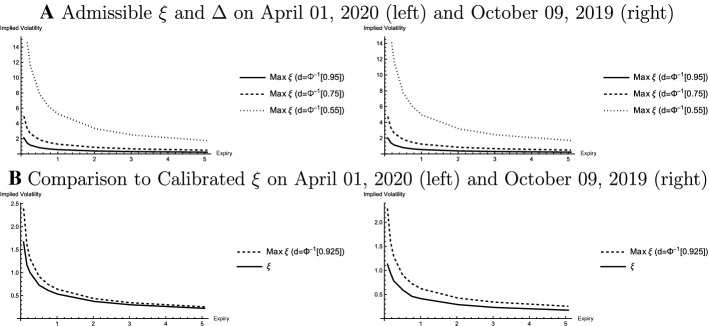
Admissible Parameters: Arbitrage-Free Approach. Panel A shows $\xi ^{max}_t$ for the estimated parameters on April 01, 2020 (left) and October 09, 2019 (right) with respect to delta and expiry for different moneyness levels in terms of the moneyness variable *d*. Panel B compares $\xi ^{max}_t$ (d=$\Phi ^{-1}(0.925)$) to the estimated volatility of implied volatility ($\xi _t^T$) for different expiries

Panel A in Fig. 4 shows the set of admissible $\xi _t$ becomes smaller for options that are further out-of-the money on both trading days. Intuitively, $\xi _t$ is positively related to the steepness of the smile. Hence, to match implied volatilities in the tails, $\xi _t$ cannot be too large. Panel B also compares the calibrated parameters $\xi _t$ to the boundary $\xi _t^{max}$ applicable to calls and puts with a forward delta of $\pm 0.075$. It turns out that the calibrated $\xi _t$ are within the set of admissible parameters even for these options. In particular, estimated parameters are not a boundary solution.

## Conclusion

This paper addresses arbitrage-free FX smile construction. The approach is nested in the model of Carr and Wu (2016) and considers the near-term dynamics of implied volatilities. The approach is directly applicable to volatility smiles on FX options market if implied volatilities are a function of non-premium adjusted deltas. Forward exchange rates and strikes are not required as an input. Arbitrage-free implied volatilities are given by the roots of a cubic polynomial and can be determined with a closed form solution. The symmetry property ensures that the prices of benchmark contracts (risk reversals and butterflies) are identified as the roots of the same polynomial while quoted ATM-volatilities can be matched by construction. A numerical application compares volatility smiles of the Arbitrage-Free Approach and the Vanna Volga Approach. Two scenarios are considered including a stress scenario and a normal trading day. For both scenarios differences are observed when smiles are extrapolated to the wings. These differences are more substantial in the stress scenario. Interpolation of implied volatilities yields similar results. When the near-term volatility is large, the smile may become too steep to price deep out-of-the-money put options.

A potential extension of this research would be to explore how well the proposed approach is able to fit the time series of volatility smiles and surfaces. Moreover, from a practical point of view it would be interesting to assess the hedging performance of the approach also in comparison to other smile construction procedures. These questions are, however, beyond the scope of this paper, which is on smile construction. They are left for future research.

## A Appendix: Localization of the roots of the cubic polynomial

Decartes’ rule of signs implies that the cubic polynomial given in Eq. (13) has exactly one positive real root and two negative real roots if and only if the discriminant *D* defined in Eq. (17) is negative and the sequence of the coefficients $\{\alpha ,\beta ,\gamma ,\delta \}$ has exactly one change of sign. The coefficient $\delta$ is positive by construction. If $\theta _t$ is identified as in Eq. (16) then the coefficient $\beta$ simplifies to

$$\begin{aligned} \beta =\xi _t^2 d^2\tau -1. \end{aligned}$$

Two cases can be distinguished:

**Case 1 **($\beta < 0$): This case implies that

22 $$\begin{aligned} \xi _t^2<\frac{1}{d^2 \tau }. \end{aligned}$$

Note that this expression is independent of the sign of *d*. If the coefficient $\rho _t$ is negative then $\gamma$ is positive if *d* is negative. This implies that $\alpha$ is negative as well. The sequence of signs is $\{-,-,+,+\}$ implying exactly one positive real root by Decartes’ rule.

If the coefficient $\rho _t$ is positive then the coefficient $\gamma$ is negative if *d* is also negative. Then, the coefficient $\alpha$ is negative. The sequence of signs is $\{-,-,-,+\}$ and there is exactly one positive real root.

**Case 2** ($\beta > 0$): This case implies that

$$\begin{aligned} \xi _t^2>\frac{1}{d^2 \tau }. \end{aligned}$$

In this case the coefficient $\gamma$ must be positive to avoid more than one change of signs. If the coefficient $\rho _t$ is negative then $\gamma$ is positive if *d* is negative. This implies that the coefficient $\alpha$ is negative and the sequence of signs is $\{-,+,+,+\}$. In contrast, if $\rho$ is positive then *d* and $\alpha$ are positive as well. The sequence of signs is $\{+,+,+,+\}$ and there are no positive roots.

In summary, the symmetry property stated in Eq. (14) implies that if $g_d(\sigma )$ has exactly one positive root and two negative roots then $g_{-d}(\sigma )$ has exactly two positive roots and one negative root. Cases 1 and 2 imply that, if $\rho _t<0$ and $g_{d}(\sigma )$ has three real roots then these roots cannot have the same sign. If $\rho _t>0$ then a sufficient condition for at least one positive and one negative is that the inequality (22) holds, that is, $\xi _t$ must not be to large. However, $\xi _t$ is also constrained to ensure that the discriminant is negative. This is also discussed in Sect. 5.4.

## Footnotes

1 See, for example, Demeterfi et al. (1999) or Jiang and Tian (2005) for continuous processes of the underlying and Carr and Wu (2009) as well as Du and Kapadia (2012) when there is jump risk.

2 In practice, transaction and spot dates as well as expiry and delivery dates of options have to be distinguished. Details are given in Wystup (2017), pp. 26-30.

3 For sake of brevity, premium adjusted deltas are not discussed in this paper. They are addressed in an appendix to this paper, which is available upon request.

4 Other important conventions are ATM-spot (ATM-S), ATM-forward (ATM-F).

5 Premium adjusted deltas are treated in an appendix to this paper, which is available upon request.

6 See Carr and Wu (2016).

7 This is also true for the “Lognormal Variance Model” (LNV) in Carr and Wu (2011).

8 Note that for currency options considered in Carr and Wu (2011), the SRV model performs slightly worse than the LNV, empirically.

9 See also Carr and Wu (2011).

10 For simplicity, the case of exactly two real roots is not addressed since this would enlarge the set of feasible parameterizations only marginally.

11 I am grateful to an anonymous referee who inspired Appendix A.

12 Note that selecting the largest negative and the smallest positive root also implies more symmetrical smiles because the absolute value of the third root is unreasonably high in numerical applications below.

13 An in depth textbook treatment of this approach can be found in Castagna (2010).

14 See Castagna (2010) for details.

15 I am grateful to an anonymous referee for pointing this out.

16 Mid-quotes are calculated from bid- and ask-quotes. The RICs are EURXXO (ATM-volatilities), EURXXRR ($25\Delta$-risk reversal), and EURXXBF ($25\Delta$-butterfly), where XX is the expiry (e.g., for the 1 month and 1 year expiries XX is 1M and 1Y, respectively).

17 Data source for bid- and ask-deposit rates is also Refinitve Eikon. The RICs are EURXXD, where XX is the expiry (e.g., for the 1 month and 1 year expiries XX is 1M and 1Y, respectively).

18 As indicated in Table 1, the mid-quotes for $\Delta 25$-risk reversals range between 0.1% and 0.4% (-1.1% and -0.3%) across expiries on the normal (volatile) day.

19 Results are qualitatively similar for the parameters on the normal trading day.

20 There might also be a minimum positive number for the choice of $\xi _t$. This minimum is usually very small and economically not meaningful. For this reason and for the sake of brevity, this issue is not discussed in detail here.
